# MicroRNAs-Role in Lung Cancer

**DOI:** 10.1155/2014/218169

**Published:** 2014-03-13

**Authors:** Małgorzata Guz, Adolfo Rivero-Müller, Estera Okoń, Agnieszka Stenzel-Bembenek, Krzysztof Polberg, Maria Słomka, Andrzej Stepulak

**Affiliations:** ^1^Chair and Department of Biochemistry and Molecular Biology, Medical University of Lublin, Ulica Witolda Chodźki 1, 20-093 Lublin, Poland; ^2^Department of Physiology, Institute of Biomedicine, University of Turku, 20520 Turku, Finland; ^3^Department of Otolaryngology, MSW Hospital, 20-331 Lublin, Poland; ^4^Chair and Department of Gastroenterology with Endoscopic Unit, Medical University of Lublin, 20-093 Lublin, Poland

## Abstract

Regulation of gene expression is essential for normal physiological functions; thus deregulation of gene expression is common in disease conditions. One level of regulation of gene expression is performed by noncoding RNAs, among which microRNAs (miRNA) are the best studied. Abnormal expression of these molecular players can lead to pathogenic processes such as heart disease, immune system abnormalities, and carcinogenesis, to name but a few. Of a length of 18–25 nucleotides miRNAs are involved in binding partial complementary sequences within the 3′-UTR (3′-untranslated region) of the target mRNAs. Depending on the type of neoplastic transformation, miRNAs can act both as oncogenes (oncomirs) or as tumor suppressors. Because of the great importance of miRNAs, most researches focus on either their role as biomarkers or their potential as therapeutic targets. Herein, we present the review of microRNA biology, function, and tumorigenic potential with emphasis on their role in lung cancer.

## 1. Introduction

Cancer represents a heterogeneous group of diseases characterized by uncontrolled cell growth promoting tumor formation and metastasis [1]. Tumors are characterized by six essential alterations in cell physiology: self-sufficiency in growth signals, insensitivity to growth-inhibition signals, evasion of apoptosis, limitless replicative potential, sustained angiogenesis, and tissue invasion and metastasis [2, 3]. The latter two are the most deadly hallmarks of malignant tumors [4].

During the past decade, the discovery of the regulatory role of microRNAs (miRNAs) upon gene expression has extensively shed light on cancer biology [1]. In normal cells, miRNAs control genes were involved in normal rates of cellular growth, proliferation, differentiation, and apoptosis. Unsurprisingly, miRNAs who inhibit genes involved in cell cycle progression and drive terminal differentiation are often downregulated in cancer cells, while others regulating genes involved in cell cycle progression and resistance to apoptosis are overexpressed [5]. Concomitantly, miRNA loci frequently map to genomic regions that are commonly amplified or deleted in human cancers [6–10]. Indeed, many tumor cell lines as well as human tumors have been found to have widespread deregulation of miRNA expression [7, 11, 12]. Furthermore, the expression pattern of certain miRNAs correlates with diverse clinicopathological parameters [6, 12, 13] and prognosis [4, 14]. These findings highlighted the potential role of miRNAs as new diagnostic or prognostic biomarkers. There is mounting evidence that specific miRNAs have tumor suppressor or prooncogenic functions making them novel targets for cancer therapy including lung cancer [15].

Lung cancer (LC) is the leading cause of cancer-related deaths all over the world, among both men and women, with an incidence of over 200 000 new cases per year and a very high mortality rate [16]. Indeed, lung cancer is responsible for more deaths than breast, colon, and prostate tumors combined [14]. LC is comprised into two major clinicopathological categories: small-cell (SCLC) and non-small-cell lung carcinoma (NSCLC).

SCLC accounts for around 12% of all cases, it is more aggressive than NSCLC, and it frequently metastasizes [17]. NSCLC tumors consist mainly of three subtypes: adenocarcinoma (40% of lung cancers), squamous cell carcinoma (25% of lung cancers), and large cell carcinoma (10% of lung cancers). NSCLC is less aggressive and more common, accounting for at least 88% of all lung cancer cases.

## 2. miRNA Biogenesis

Discovered in the early 1990s by Victor Ambros and colleagues, miRNAs comprise an abundant class of endogenous, small noncoding RNAs 18–25 nucleotides in length that repress protein translation through binding to (often only partial) complementary target mRNAs [18]. miRNA genes are evolutionarily conserved and are located within the introns of protein-coding genes, as well as within intergenic areas [19] and transcribed as long transcripts up to 1000 nucleotides long, named primary miRNAs (pri-miRNAs), which are processed by RNase III (Rnasen) and Microprocessor complex subunit DGCR8 (DGCR8) into precursor stem-loop structures 44–180 nt long termed “pre-miRNA” [20–22].

**Figure 1:**
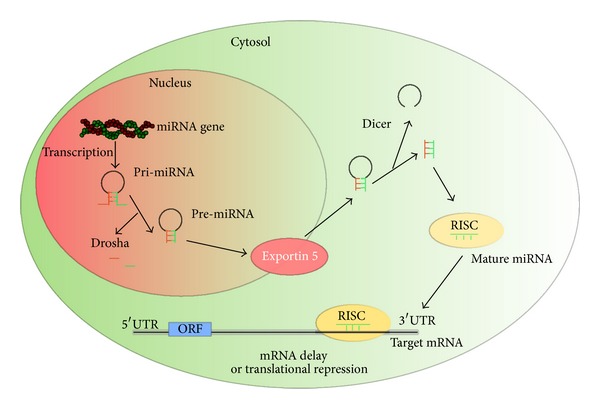
miRNA biogenesis and mechanism of action. miRNAs are transcribed by polymerase II into primary transcripts (pri-miRNAs). Pri-miRNAs are cleaved by the Drosha resulting in the formation of a hairpin precursor (pre-miRNAs). Exportin 5 transports pre-miRNAs to the cytoplasm, where Dicer processes them into miRNAs duplexes. One strand of the duplex (mature miRNA) is incorporated into the RNA-induced silencing complex (RISC) and binds to 3′-UTR of target mRNA resulting in either its degradation or translational repression.

Pre-miRNAs are transported to cytoplasm by exportin 5 (Ran GTP-dependent dsRNA-binding protein) where they are further processed to mature miRNAs by a second RNase III, named Dicer. The resulting 18–25-nucleotide mature miRNA ultimately gets integrated into the RNA-induced silencing complex (RISC). Mature miRNAs exert their regulatory effects by binding to perfect or imperfect complementary sites within the 3′-untranslated region (3′-UTR) of their mRNA targets, thereby posttranscriptionally repressing target-gene expression [23, 24]. Each miRNA may have as many as hundreds of mRNA targets [25, 26]. In general, miRNAs act in the cytoplasm, yet mature miRNAs has also been found in the nucleus [27–31] and nucleolus [32, 33] but the mechanisms of miRNAs subcellular localization and function are still not well understood [31] (Figure 1).

miRNAs were originally found to play a role in the timing of larval development in* Caenorhabditis elegans*, which lead to the identification of* lin-4 *and* let-7 *miRNAs [18, 34]. Initial understanding of miRNA-mRNA target recognition came from observations of sequence complementarity between* lin-4 *and multiple conserved sites within the gene* lin-14 *3′-UTR [35]. Notably, depending on the degree of homology with the target sequence, miRNAs induce translational repression or degradation of mRNAs [36]. At present, more than 2000 miRNAs have been characterized in humans (miRBASE, release 19, February 2013), which target at least 60% of all mRNAs [37]. In this regard, miRNAs control a wide range of biological processes including apoptosis, development, proliferation, and differentiation [15]. Given their integral role in development, it was no surprise that miRNAs were soon found to play a role in tumorigenesis [38]. Their roles in tumor development are so evident that their expression profiles can be used to classify human tumors and identify molecular signatures associated with the corresponding clinical status [9, 39].

## 3. Proliferation and Cell Cycle Are Affected by miRNAs

Uncontrolled proliferation is a crucial step in cancer progression. Recent evidence demonstrated that aberrant miRNAs expression is a critical factor influencing tumor cell growth and thus acting as either tumor suppressors or oncogenes [40]. In human lung cancer tissues and corresponding cell lines many miRNAs are usually downregulated when compared with adjacent lung tissue, as demonstrated for* let-7* [41],* miR-15a*,* miR-16* [42],* miR-34a* [43],* miR-34b* [44],* miR-125* [45],* miR-155* [46],* miR-192* [47], and* miR-486* [48], while others are overexpressed such as* miR-21* [49],* miR-194* [50], and* miR-186 *[51], when compared to normal human bronchial epithelial cells [49]. As it could be expected, in experimental conditions enforced overexpression of* miR-101* [52] and* miR-186* [51] inhibited NSCLC cell proliferation, whereas forced* miR-200* expression inhibited tumor growth and metastasis [53] Lentiviral-mediated overexpression of* miR-129* blocked proliferation of several tumor cell lines, including lung adenocarcinoma [54].

Likewise overexpression of* miR-192* inhibited cell proliferation in NSCLC cell lines A549, H460, and 95D cells and inhibited tumorigenesis in an* in vivo* model [47], while overexpression of* miR-34b* [55] and* miR-193b* [56] significantly reduced A549 cell survival. Transient introduction of* miR-34a* into NSCLC cell line A549 and SCLC cell line SBC-5 also caused complete suppression of cell proliferation [43]. Recent studies demonstrated that inhibition of individual miRNAs delay lung cancer cell proliferation, as reported for for* miR-150* [57] and* miR-223* [58]. Nevertheless, miRNAs seem to work differently depending of the cellular context; for example,* miR-34a*, which has been previously correlated to prostate cancer inhibition [59], did not influence SCLC cell viability, suggesting that* miR-34a *is unrelated to the malignant behavior of SCLC cells [60].

Given that reduced proliferation is often the result on inhibition of cell division, several studies have been focusing on the role of miRNAs of cell cycle progression. A typical eukaryotic cell cycle is divided into two basic processes: mitosis and interphase. Interphase, the period between mitoses, is the time during which cell growth and DNA replication are started in preparation for cell division. The cycle of eukaryotic cells consists of four phases: G1 phase (gap 1, between mitosis and initiation of DNA replication), S phase while DNA replication begins, and G2 phase (gap 2) during which cell growth is in progress and proteins are synthesized for the next mitosis (M phase) [61].* In vitro* studies have demonstrated that several deregulated miRNAs are involved in LC cell division.* Let-7* was the first identified miRNAs in* C. elegans* as a regulator of the timing of cell fate determination and is evolutionarily conserved across species.* C. elegans* stem cells carrying a mutant* let-7* fail to exit the cell cycle; they do not differentiate and continue to divide typical characteristics of cancer cells [34]. In humans, reduced expression of* let-7* is often present in certain types of lung adenocarcinoma and bronchioloalveolar carcinoma [62, 63]. Transient introduction of* miR-34a* into A549 and SBC-5 cell lines induced cell cycle arrest at the G1 phase [43]. Moreover,* miR-34a* and* miR-15/16* act synergistically, potentiating the impact on inhibition of G1/S progression [64]. Similarly, ectopic expression of* miR-137* in A549, NCI-H460, and NCI-H520 resulted in G1 cell cycle arrest [65].* miR-129* induced G1 phase arrest in multiple human lung adenocarcinoma cell lines, suggesting* miR-129* targeting of G1/S phase-specific regulators [54].* Pre-miR-630* arrested A549 cells in the G0/G1 phase of the cell cycle, resulting in greatly diminished sensitivity of A549 cells to the late S/G2/M cell cycle arrest [66]. In contrast, only* miR-223* was found to induce G2/M arrest in NSCLC cells [58].

The transition from one cell cycle phase to another is regulated by different cellular proteins. A crucial role is played by the cyclin-dependent kinases (CDKs), who become activated via phosphorylation at particular time-points of cell cycle. Five of them are active during the cell cycle: in G1 phase they are CDK4, CDK6, and CDK2 and in S phase it is CDK2, whereas in G and M phases it is CDK1 [67]. CDKs are stable during the cell cycle in opposition to their activating proteins, cyclins. At different phases of the cell cycle different cyclins are required. Essential for entry to G1 phase are CDK-cyclin D complexes formed by three D type cyclins (D1, D2, and D3) with CDK4 and CDK6 [68]. Another G1 phase cyclin is cyclin E that forms a complex with CDK2 and regulates progression from G1 to S phases [69], whereas cyclin A/CDK2 complex is required during S phase [70, 71]. In late G2 and early M phase, complex cyclin A with CDK1 promotes entry into M phase, and afterwards M phase is regulated by cyclin B associated with CDK1 [72, 73]. Since most of the miRNAs act on genes involved in G1/S phases of the cell cycle, their targets include Cdk2/Cdk6 and related cyclins. Indeed, CDK2 was identified as direct targets of* miR-223* in a Lewis lung carcinoma (LLC) cell line [58], and, according to gene expression and bioinformatic analyses of NSCLC cells, CDK6 was identified as a target gene of* miR-129* [54] and miRNA-214 [74]. Ectopic expression of* miR-137* in NSCLC cell lines (A549, NCI-H460, and NCI-H520) downregulated Cdc42 and Cdk6 and induced G1 cell cycle arrest, resulting in decreased cell growth* in vitro* and* in vivo* [65]. Likewise, CDK2 and CDK6 were each directly targeted by* miR-186* and restoring their expression reversed* miR-186*-mediated inhibition of cell cycle progression [51]. Whereas, cyclin D1 (CCND1) and CDK6 seem to be targets of* miR-34a* that results in a G1 cell cycle arrest [75]. CCND1 expression is controlled by* miR-193b* and* miR-206* [76] in the A549 cell line [56], suggesting an involvement of* miR-206* in tumorigenesis [76]. In NSCLC cells cyclins D1, D2, and E1 are directly regulated by physiologic concentrations of* miR-15a*/*miR-16*, showing inverse correlation between* miR-15a*/*miR-16* and CCND1 expressions [64]. Direct evidence for the effect of* miR-34c* on cyclin E regulation was provided by using cells extracted from a mouse model overexpressing cyclin E, who develops multiple pulmonary adenocarcinomas, and human LC cell lines transfected with* miR-34c* resulting in repression of cell proliferation [77].

CDKs activity can be regulated by cell cycle inhibitory proteins (CKI) that bind to CDKs or CDK-cyclin complexes. There are two families of CDKIs: INK4 family (p15, p16, p18, and p19) and Cip/Kip family (p21, p27, and p57) [78]. Members of these families form stable complexes with CDK4/6 before cyclin binding, which prevents the activation and formation of CDK-cyclin D and CDK1-cyclin B complexes [79, 80]. To date, there are no reports describing miRNA-regulation of INK4 family members. However, it was demonstrated that several miRNAs affect cell cycle progression at different phases of cell cycle acting on members of Kip/Cip family.* Let-7a* elevates p21 (CDKN1A) levels via UHRF2 (ubiquitin-like with PHD and ring finger domains 2) inhibition and suppresses the growth of A549 lung cancer cells [41], whereas* miR-128-2* posttranscriptionally targets E2F5 and leads to the abrogation of its repressive activity on p21 (waf1) transcription in a human NSCLC cell line (H1299) [81]. Likewise,* pre-miR-630* expression arrests A549 cells in the G0/G1, which correlates with the increased levels of p27 (CDKN1B), another cell cycle inhibitor [66]. In contrast, direct inhibition of p27 by at least two micrRNAs,* miR-221*,* miR-222,* and* miRNA-194* results in enhanced cell survival to the NSCLC cells [82].

Another well-known example of a miRNA target gene is the tumor suppressor p53, the most frequently mutated gene in human cancers. p53 protein regulates genes and their products associated with cell cycle progression, apoptosis, DNA repair, or genomic stability [83]. As miRNAs control p53, the opposite is also possible as p53 affects the expression of several miRNAs. The* miR-34* family members are downstream transcription targets of p53; thus* miR-34* is reduced in p53 mutant tumors. Exogenous addition of* miR-34* to tumor cells reduced proliferation and invasiveness* in vitro* and tumor formation* in vivo* [84]. Besides* miR-34a*, other miRNAs (*miR-184*,* miR-181a,* and* miR-148*) are also regulated by p53 protein* in vitro* and* miR-150* expression was correlated with p53 in human NSCLC patients tissues samples [85]. However, the mechanism how p53 affects the expression of these miRNAs is still unknown. Studies on the restoration of p53 pathway may provide effective approaches for anticancer therapy. p53 expression was higher when cancer NSCLC cells were treated with anti-*miR-150* expression vector that indicates that the upregulation of p53 contributes to cancer growth [86].* miR-150* targets p53 in NSCLC cell lines (SPCA-1, A549, HCC827, 95-D, and BEAS-2B), which in turn regulates the expression of various tumor-suppressor miRNAs participating in cell cycle progression [85]. Earlier reports suggested that some small noncoding RNAs take part in the regulation of p53 expression [87]. Indeed, p53 expression was found to be downregulated by* miR-98*,* miR-453* [88], and* miR-378* [89]. In contrast, wild type p53 mRNA and protein were increased by* miR-125a* overexpression [45] or activated by* miR-29* via p85*α* (PI3K) and CDC42 [90]. Thereby, the importance of miRNAs to be regulated and regulating p53 in tumorigenesis are highlighted, indirectly involved in the cell cycle progression. Accordingly, targeted deletion of* miR-31* results in the repression of nonsmall cell lung cancer cells growth and* in vivo* tumorigenicity due to higher expression of LATS2 (human large tumor suppressor 2) and PPP2R2A (PP2A regulatory subunit B alpha isoform) [91].* LATS 2* encodes a putative serine/threonine kinase that exerts tumor-suppressive effects by inhibition of the G1/S cell cycle transition [92]. PPP2R2A is a regulatory subunit of protein phosphatase 2 (PPA2), a highly conserved serine/threonine phosphatase, which exerts its function in tumorigenesis by negative regulation (dephosphorylation) of cell cycle regulators such as cell division control protein 2 homolog (CDC2) and M-phase inducer phosphatase 3 (CDC25C) [93]. Such modulation of the LATS2/PPP2R2A pathway by* miR-31* constitutes a novel growth regulator in lung cancer [94].

## 4. miRNAs Regulating Apoptosis

Defective programmed-cell death determines a major causative factor in the development and progression of tumor. Central biochemical machinery of apoptosis underlies the activity of the caspases, an aspartate-specific cysteine proteases which cleave to activate/inactivate targets within the cell. The sequence of events culminating in the activation of caspases is categorized into extrinsic and intrinsic pathways [95]. The extrinsic pathway can be mediated by one or several death receptors when bound by the appropriate ligand [96]. The death receptor family also known as the tumor necrosis factor (TNF) receptor family includes TNF, Fas (CD95), and TRAIL 1–5 (TNF-related apoptosis-inducing ligand) receptors. A still growing number of experimental data suggest that miRNAs have an inhibition effect on apoptosis-related molecules, that is, TNF-*α* and mainly TRAIL. TNF-*α* is a target for* miR-19a* and a member of* miR-17-92* cluster, and this cluster is often found overexpressed or amplified in many malignant tumors including lung cancers [97], suggesting that* miR-19a* could be a novel target to sensitize cancer cells to apoptosis [97]; however this issue requires further studies.

As mentioned above,* miR-34a* and* miR-34c* are downregulated in NSCLC cell lines, and overexpression of these two noncoding molecules is linked to TRAIL-induced apoptosis, reducing the invasive capacity of NSCLC cells [98]. In addition, ectopic expression of* miR-212* increases TRAIL-induced cell death in lung cancer cells [99]. However, several tumors, including lung cancer, have developed resistance for TRAIL induced apoptosis using miRNA depending mechanisms, such as* miR-494a*, whose downregulation made the A549 cell line more sensitive to TRAIL-induced apoptosis [100]. Two additional miRNAs,* miR-221* and* miR-222,* impair TRAIL-induced apoptosis, thus transfection blocking these miRNAs with* anti-miR-221* and* anti-miR-222* results in TRAIL-sensitivity in NSCLC, a mechanism involving the downregulation of p27, demonstrating that high expression of* miR-221* and* miR-222* maintains the TRAIL-resistant phenotype [101]. In turn,* miR-130a* was able to reduce TRAIL resistance in NSCLC cells through c-Jun downregulation of* miR-221* and* miR-222* expression [82].

The intrinsic apoptosis pathway is characterized by the rapid release of cytochrome c (CYCS) from the mitochondrial intermembrane space into the cytosol. The releasing mechanism of CYCS requires permeabilization of the outer mitochondrial membrane, with a loss of membrane potential and the presence of members of pro- and antiapoptotic Bcl-2 proteins [102, 103]. In the cytosol CYCS binds an adapter protein Apaf-1 (apoptotic protease activating factor 1) that in the presence of dATP/ATP forms Apaf-1 multimer recruiting procaspase [104, 105]. Procaspase-9 becomes activated and in turn it activates caspase-3, an effector caspase involved in the dismantling of the cell structures during apoptosis. Effector caspases, caspase-3, caspase-6, and caspase-7, when activated cleave cytoskeletal and nuclear proteins [106].

There are miRNAs known to regulate proapoptotic (BAK, Bax, Bcl-rambo, Bcl-xs, BOK/Mtd, and BH-3 proteins) as well as antiapoptotic (Bcl-2: Bad, BID, Bik/Nbk, BIM, BLK, Bmf, Hrk/DP5) or antiapoptotic (Bcl-2, Bcl-xL, Bcl-w, Mcl-1, Bcl-10, and Bcl-2 related protein A1). In experimental conditions, ectopic expression of* miR-503* causes reduced expression of antiapoptotic Bcl-2 protein in NSCLC A549 cells [107]. Enforced* miR-181b* [108],* miR200b/c-429* cluster [109], and* miR-497* [110] expression downregulated Bcl-2, suggesting that Bcl-2 was the target gene of these miRNAs in NSCLC cell lines. Different miRNAs have also been shown to affect expression and function of other antiapoptotic proteins of the Bcl-2 family. For example,* Let-7c* significantly reduced luciferase activity of Bcl-xL 3′UTR-based reporter, simultaneously reducing Bcl-xL protein levels [111], whereas* miR-125b* expression induced spontaneous apoptosis in various cell lines derived from lung cancer and sensitized cancer cells to different apoptotic stimuli, probably by suppressing the antiapoptotic molecules Mcl-1 and/or Bcl-w, suggesting that* miR-125b* downregulation facilitates tumor development [112]. Likewise, several miRNAs modulate expression of proapoptotic members of Bcl-2 family. Luciferase reporter assays demonstrated that in lung cancer cells* miR-221/222* comodulated PUMA (p53 upregulator of apoptosis) expression, by directly targeting the binding site within its 3′-UTR [113]. Overexpression of* miR-494* [100] and* miR-17* [114] downregulates BIM protein (Bcl-2-like 11). Similarly,* in vitro* and* in vivo* studies showed the inhibited expression of BIM by* miR-30b*,* miR-30c*,* miR221*,* miR-222*,* miR-103*, and* miR-203* in NSCLC cells [115]. Silencing of* miR-155* elevated expression of the Apaf-1 proteins, whereas Apaf mRNA levels were unchanged [46]. Mitochondrial/postmitochondrial steps of the intrinsic pathway of apoptosis, including Bax oligomerization, mitochondrial transmembrane potential dissipation, and the proteolytic maturation of caspase-9 and caspase-3, were modulated by* pre-miR-181a* and* pre-miR-630* in A549 lung cancer cell line [66].

Several studies have investigated the role of miRNAs in the induction of apoptosis through caspase activation via extrinsic and intrinsic pathways, finding that a number of miRNAs may rescue lung cancer cells from caspase-8 activation in death receptor-mediated apoptosis. These miRNAs included members such as* miR-17*,* miR-135,* and* miR-520* [116]. On the other hand, it was confirmed that* miR-186* promotes apoptosis by targeting caspase-10 in A549 cells [117].

A number of miRNAs were shown to interfere in apoptosis by affecting expression and activation of effector caspases. This is the case for* miR-1* in A549 lung cancer cells which enhances activation of caspase-3 and caspase-7 [118]. The same was demonstrated for* miR-15a-3p*, a novel member of the proapoptotic miRNA cluster* miR-15a/16* [119]. Caspase-3, together with caspase-7, is also activated by inhibition of* miR-133a/b* and* miR-361-3p *in 95D lung cancer cell line [120]. Apart from caspase-3, the proteolytic activation of caspase-9 is modulated by* pre-miR-181a* and* pre-miR-630* in A549 cell line [66]. Cells viability are also reduced by overexpression of* miR-192* by caspase-7 activation in* in vivo* and* in vitro* studies [47], demonstrating that the modulation of caspases activity is a promising therapeutic target.

## 5. miRNAs Regulate Lung Cancer Angiogenesis

As tumors expand, the distance between the centre of the tumor and blood vessels becomes too large so the centre turns hypoxic. The sustained cell growth in the tumor requires that oxygen and metabolites can be delivered to the tumor cells and thus the formation of new blood vessels. Hypoxia triggers a series of molecular events that result in the activation of angiogenesis (see below) so these two mechanisms are closely related [121].

Studies focused on the role of specific miRNAs in the regulation of angiogenesis are increasingly being performed. The growth of the new blood vessels is necessary for solid tumors to keep growing and spreading in a process called angiogenic switch. In lung tumor tissues where angiogenesis is taking place with higher microvessel density, there is a decreased expression of* let-7b* and* miR-126*, as compared to normal lung tissues, suggesting that low expression of these miRNAs could have antiangiogenic role in lung cancer [122], whereas* miR-16* overexpression reduced the ability of endothelial cells to form blood vessels* in vivo* [123].

Many miRNAs display organ-specific expression patterns suggesting cell-type-specific functions [124–126]. Increasing evidence indicates that miRNAs are important regulators of tumor angiogenesis as well as hypoxic responses. Hypoxia, a major hallmark of tumorigenesis, modulates activity of hypoxia-inducible factor 1 (HIF-1) [127]. HIF-1-*α* was identified as a direct target for* miR-17-92* by mass spectrometry analysis [128]. Likewise,* miR-519c* was shown as a hypoxia-independent regulator of HIF-1-*α* acting through the direct binding to the HIF-1-*α* 3′UTR. Overexpression of* miR-519c* resulted in a significant decrease of HIF-1-*α* and the reduction of tumor angiogenesis [129]. Conversely,* miR-210* expression is regulated by hypoxia-mediated HIF-1-*α* via an HRE (hypoxia-responsive element) in its promoter [130], which in turn stabilizes HIF-1-*α* through positive regulatory loop [131]. HIF-1-*α* induces the expression of different angiogenic growth factors, including vascular endothelial growth factor (VEGF) and platelet-derived growth factor (PDGF), acting on its respective receptors and promoting formation of new capillaries of tumor [132–134]. It seems that VEGF expression in lung cancer could be regulated by several miRNAs. The* Vegf* gene produces several alternative splicing forms, VEGF_121_, VEGF_145_, VEGF_165_, VEGF_183_, VEGF_189_, and VEGF_206_, where each of them is involved in different properties and functions, such as association with cell surface or extracellular matrix, and has effects on the diameter of the vessel during tube formation [135]. VEGF receptors (VEGF-R1, 2, and 3) are expressed at high levels particularly during embryogenesis. In adults, VEGF-R1 and VEGFR2 are mainly expressed in the blood vascular system in adults, whereas VEGF-R3 is restricted to the lymphatic endothelium [136]. VEGF-A is directly downregulated by miR-126 in NSCLC cells, while miR-26a expression results in its upregulation through a yet unknown mechanism [137, 138]. Additionally, the VEGFR2 is directly regulated by* miR-200c* in A549 cells [139].

The angiogenesis process in lung cancer is also regulated by many cytokines all of which are regulated by miRNAs; these cytokines include the fibroblast growth factors (FGF) as well as interleukin-8 (IL-8). FGFs are composed of 22 members that bind and activate the four FGF receptor family members (FGFR1-4); these ligands include the basic FGF (bFGF and FGF2),which plays important role in NSCLC tumor proliferation and angiogenesis [137, 140–143]. In lung tumor tissue of patients with detectable nodal metastasis, FGF seems to be specifically regulated by* miR-155*, while other angiogenic growth factors (VEGF-A, PDGF-B, and HIF-1-alpha) are not [144]. Likewise, angiogenesis-related IL-8 levels [145, 146] often elevated in tumor samples of patients with NSCLC [147] can be regulated by* miR-200* which also targets growth regulated alpha protein (CXCL1) secreted by the tumor endothelial and cancer cells. Indeed, the therapeutic potential of* miR-200* was put to the test by delivery of* miR-200* into the tumor endothelium which resulted in marked reduction in metastasis and angiogenesis, as well as vascular normalization [148]. In contrast, overexpression of* miR-378* enhances the expression of IL-8 and consequently increases stimulation of endothelial cells in NSCLC patients [89].

Other angiogenesis-related proteins are also regulated by miRNAs, for example, ectopic expression of* miR-381* reduced at mRNA and protein level a putative stem cell gene ID1 (inhibitor of differentiation 1), involved in invasion and angiogenesis, and thus significantly decreased lung cancer cells migration and invasion [149], whereas ectopic expression of* miR-26a* enhances lung cancer metastasis potential via modulation of metastasis-related genes, among them matrix metallopeptidase 2* (MMP-2)* [150].

## 6. microRNAs as Next-Generation Biomarkers for Lung Cancer

The diagnosis of lung cancer at its early stage is essential for improving the effect of treatment and survival rate of patients. Thus, there is a great need for easy detectable biomarkers determining existence of lung tumors or monitoring disease progression in cancer-bearing patients [151]. Thanks to the advances in sequencing technologies (i.e., next-generation sequencing, NGS) and bioinformatics tools it is possible to screen large data sets of miRNA expression patterns from normal and malignant human cells in order to find novel and reliable biomarkers.

In fact, it has been reported that human serum/plasma contains large amounts of stable miRNAs and their expression patterns in body fluids have a great potential as biomarkers of many diseases [152, 153]. More importantly, the circulating miRNAs levels correlate to cancer progression, therapeutic response, and patient survival. MicroRNAs expression profiles might be diagnostic and prognostic markers for many types of cancers, including lung cancer [154].

One of the pioneering reports using quantitative reverse transcription PCR (qRT-PCR) assays found higher expression levels of four miRNAs (*miR-486, miR-30d, miR-1*, and* miR-499*) in serum of patients with NSCLC. The results demonstrated the similarity between the circulating exosomal miRNA and the tumor-derived miRNA patterns, as well as the clear difference in total exosome and miRNA levels between lung cancer patients and healthy controls [152]. Later on, Rabinowits and collaborators reported the presence of 12 miRNAs in serum of lung adenocarcinoma-bearing patients,* miR-17-3p, miR-21, miR-106a, miR-146, miR-155, miR-191, miR-192, miR-203, miR-205, miR-210, miR-212*, and* miR-214*, in circulating exosomes suggesting that circulating exosomal miRNA might be useful in a screening test for patients with lung adenocarcinoma [155].

The profiles of circulating miRNAs were also compared in patients with lung squamous cell carcinoma before and after tumor removal. The levels of five miRNAs* miR-205, miR-19a, miR-19b, miR-30b*, and* miR-20a* were downregulated in plasma of patients 7–10 days after tumor removal. Another recent study confirmed that* miR-205* level consistently decreased in the serum of lung cancer patients after tumor resection [156]. Large-scale analysis on NSCLC indicated that, among 46 miRNAs differentially expressed in PMBC (peripheral blood mononuclear cells), 42 miRNAs were downregulated after lung cancer resection. Significant changes were related to* let-7c, miR-34a, miR-202, miR-769-5p,* and* miR-642* expression levels [157]. Conversely, Leidinger et al. only found slightly differences in expression pattern of* miR-34a* before and after NSCLC surgery and* let-7c* upregulation after surgery, whereas* miR-202* and* miR-769p* were not expressed neither in pre- nor in in postsurgery samples in limied group of patient's plasma. However, follow-up of the fate of the plasma miRNome of lung cancer patients, starting prior to surgery and ending 18 months after surgery, shows specific fluctuating miRNA patterns with a significant correlation between miRNAs expression level and time distance from surgery [158]. Therefore, it seems that miRNAs are released or leaked by tumor cells and circulate in stable form in the bloodstream, although the mechanism of miRNAs release is not well understood [159, 160]. In the experimental conditions,* miR-451* and* miR-205* are secreted to the medium by cultivated A2182 lung cancer cell line. Likewise,* miR-30b *was found to be excreted by A549 lung adenocarcinoma cells, proving that cancer cells can release miRNAs to the environment [161].

miRNAs are also present in sputum, an aspect that allowed Yu and collaborators to distinguish between patients with lung adenocarcinoma and healthy controls. Analyzing several miRNAs they showed that four of them,* miR 486, miR-21, miR-200b*, and* miR-375,* in combination produced the best prediction in distinguishing lung adenocarcinoma patients from normal subjects with 80.6% sensitivity and 91.7% specificity [162]. Furthermore, they identified another three miRNAs in sputum,* miR-205, miR-210*, and* miR-708,* that characterize squamous cell lung carcinoma patients [163], demonstrating that miRNAs could be specific for histologic type of lung cancer. Performing similar type of study Lee and collaborators investigated the expression of a panel of 7 miRNAs (*miR-21, miR-29b, miR-34a/b/c, miR-155*, and* let-7a*) in 31 SCLC tumors, 14 SCLC cell lines, and 26 NSCLC cell lines and observed significantly lower* miR-21, miR-29b,* and* miR-34a* expression in SCLC cell lines [60]. However, miRNA levels could not distinguish between patients with benign lung disease and lung cancer patients, although the levels of* miR-10b, miR-141,* and* miR-155* were significantly higher in lung cancer patients than those in patients with benign disease [164].

Nevertheless, tissue samples examination using qPCR or microarray analysis showed significant differences in the amounts of particular miRNAs between lung cancer tissues (LCT) benign disease and tumor adjacent or normal lung tissues (NLT). Twenty-seven miRNAs were observed to be deregulated greater than twofold in LCT compared with NLT by microarray analysis [165]. In detail, the expression levels of* miR-21* [165] and* miR-155 *[46, 119] were significantly higher in LCT than in adjacent normal tissue.* MiR-205* was also overexpressed in NSCLC [166, 167], which is supported by new data confirming higher levels of* miR-205* in tissues and serum from NSCLC and SCLC patients [168].

On the other hand, most of the analyzed miRNAs expression in clinical samples showed lower levels in lung cancer tissues compared to adjacent or noncancerous lung tissues.* MiR-15a/miR-16* were frequently deleted or downregulated in squamous cell carcinomas and adenocarcinomas of the lung [64]. Likewise, the expression of* miR-34b* was lower in NSCLC tissues compared to that in pericarcinous tissues of lung cancer [44]. The same was reported for* miR-150* [169],* miR-186* [51],* miR-192* [47],* miR-193b* [56],* miR-486* [48], and* miR-3940-5p* [169] suggesting that loss of these miRNAs may be important in lung cancer development. However, contradicting reports suggest the association of miRNAs expression and tumor size. The expression of* miR-150* in T2 stage tissue samples was higher than in T1 stage [85], whereas other authors presented data showing that* miR-150* was downregulated in a subgroup of patients with tumor diameter more than or equal to 3 cm as well as in clinical stages III and IV [169].

One report showed that* miR-210* was overexpressed at late stages of NSCLC [170], as well as* miR-574p,* previously considered a serum-based biomarker for early stage NSCLC [171].

Late stages of lung cancer are often associated with the presence of nodal and distance metastases. In lung cancer patients high serum* miR-10b* values associated with lymph node metastasis [164]. Likewise,* miR-26a* expression level was higher in lymph node metastasis tumor tissues than in primary tumor tissues [150], suggesting direct involvement of* miR-26a* in the metastatic potential of lung cancer cells. In contrast, the lower* miR-34b* expression in cancer tissue was correlated with higher lymph node metastasis [44], and* miR-126* was a significant negative prognostic factor in lymph node-positive subgroup of patients [137]. Interestingly, several miRNAs were found to be significantly differentially expressed between primary lung tumors and the metastatic tumors to the lung from other localizations. Two of them,* miR-126* and* miR-182,* represent potential biomarkers for distinguishing between primary and metastatic lung tumors, reflecting lung tissue specificity [172].

In the clinical specimens of NSCLC* miR-194* expression was also associated with metastasis. Consequently, overexpression of* miR-194* in lung cancer cell lines resulted in suppressing metastasis of lung cancer cells, while inhibiting its expression through “miRNA sponge” promotes cancer cells to metastasize [50]. Similarly, forced* miR-200* expression inhibits tumor growth and metastasis, whereas in lung cancer patients* miR-200* levels were suppressed in metastasis-prone tumor cells and predicted poor prognosis [53].

High* miR-16* expression levels were associated with shorter disease-free survival (DFS) or overall survival (OS) of lung cancer patients [173]. Likewise, Markou and coworkers found that overexpression of mature* miR-21* was an independent negative prognostic factor for overall survival in NSCLC patients [174], and overexpressed* miR-155* in lung cancer cells correlated with poor patients prognosis [175]. In contrast, overexpression of* miR-519c* was observed in cancer patients with better prognosis [129], whereas low levels of* miR-34a* expression in cancer tissue correlated with high probability of relapse [176], while low levels of* miR-186* in NSCLC cells correlated with shorter patients OS [51]. Similarly, low expression of* let-7b *and* miR-126* correlated with worse progression-free survival and overall survival [122], and coexpression of* miR-126* and VEGF-A had a significant prognostic impact with 5-year survival of lung cancer patients [137].

But not all independent reports support the notion that miRNAs can be used as prognosis markers. Expression of* miR-21, miR-29b, miR-34a/b/c, miR-155,* and* let-7a* was determined from 639 IALT (the International Adjuvant Lung Cancer Trial) patients with NSCLC, finding no significant association between any of the tested miRNAs and survival, with the exception of* miR-21* for which a deleterious prognostic effect of lowered expression was suggested [177]. Likewise, SCLC-bearing patients-*miR-21, miR-29b, miR-34a/b/c, miR-155*, and* let-7a* were unrelated to clinical characteristics and were neither prognostic in terms of overall survival or progression-free survival nor predictive of treatment response in SCLC tumors and cell lines or NSCLC cell lines [60]. The observed discrepancies could result from different methods of miRNAs isolation, storage conditions, type of biological material (serum, plasma, and cancer tissues), or detection methods.

Recent data indicate that not only different miRNAs patterns but also miRNA-related polymorphisms may be associated with NSCLC patients clinical outcomes [178], opening new field for miRNAs investigations in relation to lung cancer progression.

## 7. Concluding Remarks

Many studies have demonstrated that miRNAs have unique expression profiles in different types of tissues. Among different lung cancer biomarkers miRNAs are the most promising because of remarkable stability, cancer-type specificity, and the presence in body fluids. The relationship between different miRNAs profiles in body fluids and tumor progression, presence of metastasis, and patients clinical prognosis rise the possibility for using miRNAs as biomarkers. However, the identification for specific miRNAs unequivocally associated with clinicopathological features of lung cancer is still work in progress. The growing number of bioinformatic and biochemical analyses opens up new possibilities for the discovery of such biomarkers, especially that the number of existing miRNAs is still growing, bringing new promising targets in the battle against cancer. The role of miRNAs as biomarkers in the detection of tumorigenesis is of vital importance, since lung cancer has a poor prognosis. So extensive and sensitive studies in this area are necessary to be successful in prospective clinical applications.
